# Protective effects of skin-derived precursor cell exosomes against UVB-induced skin photodamage

**DOI:** 10.1038/s41598-026-48604-1

**Published:** 2026-04-28

**Authors:** Ke Xian, Lumei Liu, Xin Huang, Qian Tang, Jixiang Xu, Zongjunlin Liu

**Affiliations:** 1 Department of Dermatology, The Affiliated Hospital of Southwest Medical University, Luzhou, 646000 China; 2 Clinical Medical College, Southwest Medical University, Luzhou, 646000 China

**Keywords:** Skin photodamage, SKPs-derived exosomes (SKPs-Exo), 3D skin model, Oxidative stress (OS), Nrf2, HO-1, Cell biology, Diseases, Drug discovery, Medical research, Molecular biology

## Abstract

Skin photodamage affects appearance and increases cancer risk, yet existing treatments are inadequate. Our prior data indicate that skin-derived precursors (SKPs) protect against photodamage via exosome-mediated signaling, though the mechanism is unclear. Exosomes are stable stem cell regulators, but SKPs-derived exosomes (SKPs-Exo) are underexplored in photodamage. To assess SKPs-Exo’s therapeutic potential and mechanism in UVB-induced skin damage using mouse and 3D skin models. SKPs-Exo were isolated via ultracentrifugation and characterized by TEM, NTA, and WB. UVB-irradiated mice and 3D skin models received varying SKPs-Exo doses. We evaluated skin damage, apoptosis (TUNEL), oxidative stress (ROS, MDA, GSH, SOD), inflammation (IL-1β, IL-6, TNF-α), and Nrf2, HO-1, BACH1, and NF-κB expression. SKPs-Exo (30–200 nm) expressed CD9, CD63, and TSG101. UVB-induced damage, apoptosis, oxidative stress, and inflammation were dose-dependently reduced by SKPs-Exo, which restored antioxidants, suppressed inflammation, and modulated Nrf2/HO-1 and BACH1/NF-κB pathways (*P* < 0.05 or *P* < 0.01). SKPs-Exo alleviate UVB-induced skin damage by reducing apoptosis, oxidative stress and inflammation, likely via activating the Nrf2/HO-1 pathway and suppressing the BACH1/NF-κB pathway, providing a potential therapeutic direction for skin photodamage.

**Supplementary Information:**

The online version contains supplementary material available at 10.1038/s41598-026-48604-1.

## Introduction

**Fig. 1:**
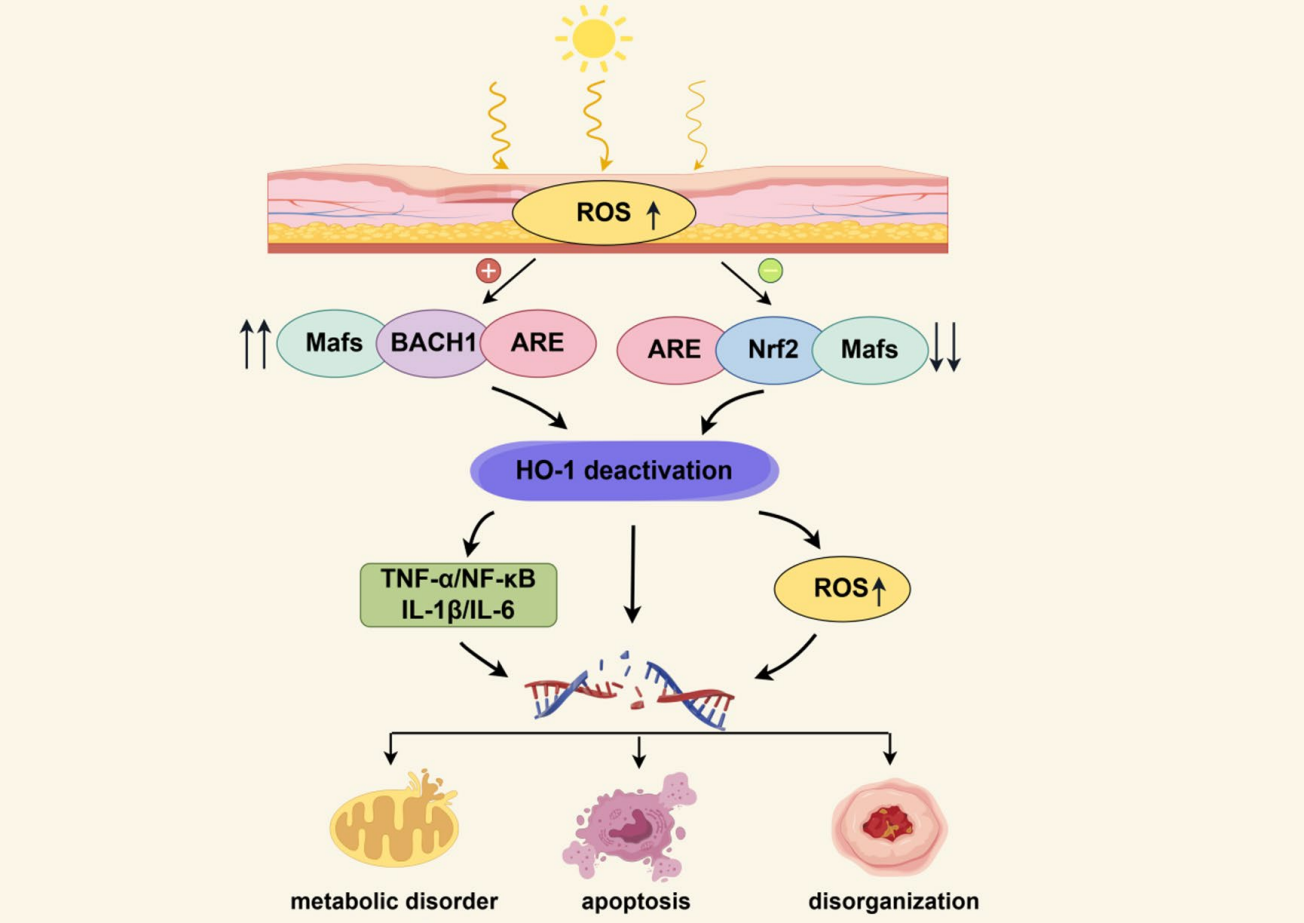
The specific pathogenesis of skin photodamage.

Skin photodamage is a specific damage caused by excessive or long-term (UV) radiation, both affecting the appearance and mentality of individuals, even possibly developing to skin cancer^1,3^. At present, it unanimously agrees that the pathogenesis of skin photodamage is inseparable from UV-induced reactive oxygen species (ROS) as well ROS-mediated oxidative stress, especially involving the dysregulation of transcription factor NF-E2-related factor (Nrf2), heme oxygenase-1 (HO-1), and transcription factor BTB-CNC homolog 1 (BACH1) signaling^3–6^. As a couple of contradictory-function proteins, Nrf2 and BACH1 crucially work in skin photodamage through recognizing the same site to regulate HO-1^6–8^. Exposure to UV ramps up ROS in skin, inducing the formation of BACH1/Mafs heterodimers that block Nrf2 from activating antioxidant signals, HO-1 in particular^5–7^. This serial alteration deactivates the skin’s antioxidant system and fuels oxidative stress (OS) that further triggers inflammatory cascades dominated by TNF-α, NF-κB, IL-1β and IL-6. Above reactions wreak havoc on cellular DNA, proteins, and lipids, consequently escalating into cell necrosis, metabolic disturbance and structural disruption of tissues, ultimately initiating skin photodamage^5,9,10^ (Fig. 1). Thus, delving into novel strategies to protect the skin from photodamage, with a particular focus on key targets like Nrf2 and HO-1, is extremely significant.

Although a variety of treatments have been tried for skin photodamage, e.g. sunscreen external use, antioxidants application, cytokines injection and photoelectric techniques, etc., few have yielded lasting satisfactory effects^1,11^. Recently, stem cells and their exosomes get more attention owing to their diverse therapeutic potentials and inherent advantages; notably, exosomes have emerged as a prime candidate for cell replacement therapy^12–15^. In our early studies, by targeting crucial regulators Nrf2 and HO-1, we found that dermal stem cells, i.e. skin-derived precursors (SKPs), alleviated the visual and histopathological signs well with a concurrent rise in Nrf2 and HO-1 expression following the transplantation into UV-irradiated models both in vivo and in vitro, the underlying mechanism possibly involving the paracrine channel namely exosomal pathway^17,18^. Increasing studies, indeed, have confirmed that the function of stem cells depends on paracrine structures–exosomes; particularly SKPs-derived exosomes (SKPs-Exo) are highly favored because of their qualities of high stability, wide source, long-term storage and easy extraction^18–20^. Nowadays, SKPs-Exo are making their way into medical applications, including treatments for skin injury conditions, neurological complaints, OS-related diseases, wound healing, tissue regeneration, drug delivery, etc.^19–21^. In our prior research, for instance, we encapsulated active proteins through using Exo to enhance their intracellular uptake efficiency and targeting capabilities, thereby significantly improving the therapeutic efficacy of these active proteins^22–24^. Nevertheless, the capability of SKPs-Exo for treating skin photodamage has rarely been studied. So, it is essential to illuminate the precise mechanism of SKPs and how their exosomes combat skin photodamage. Accordingly, the present study employed an animal model and a three-dimensional (3D) model of skin photodamage to investigate the potential for SKPs-Exo mitigating photodamaged symptoms (Supplementary Table 4).

## Results

### Characteristics and morphology of SKPs-Exo

**Fig. 2:**
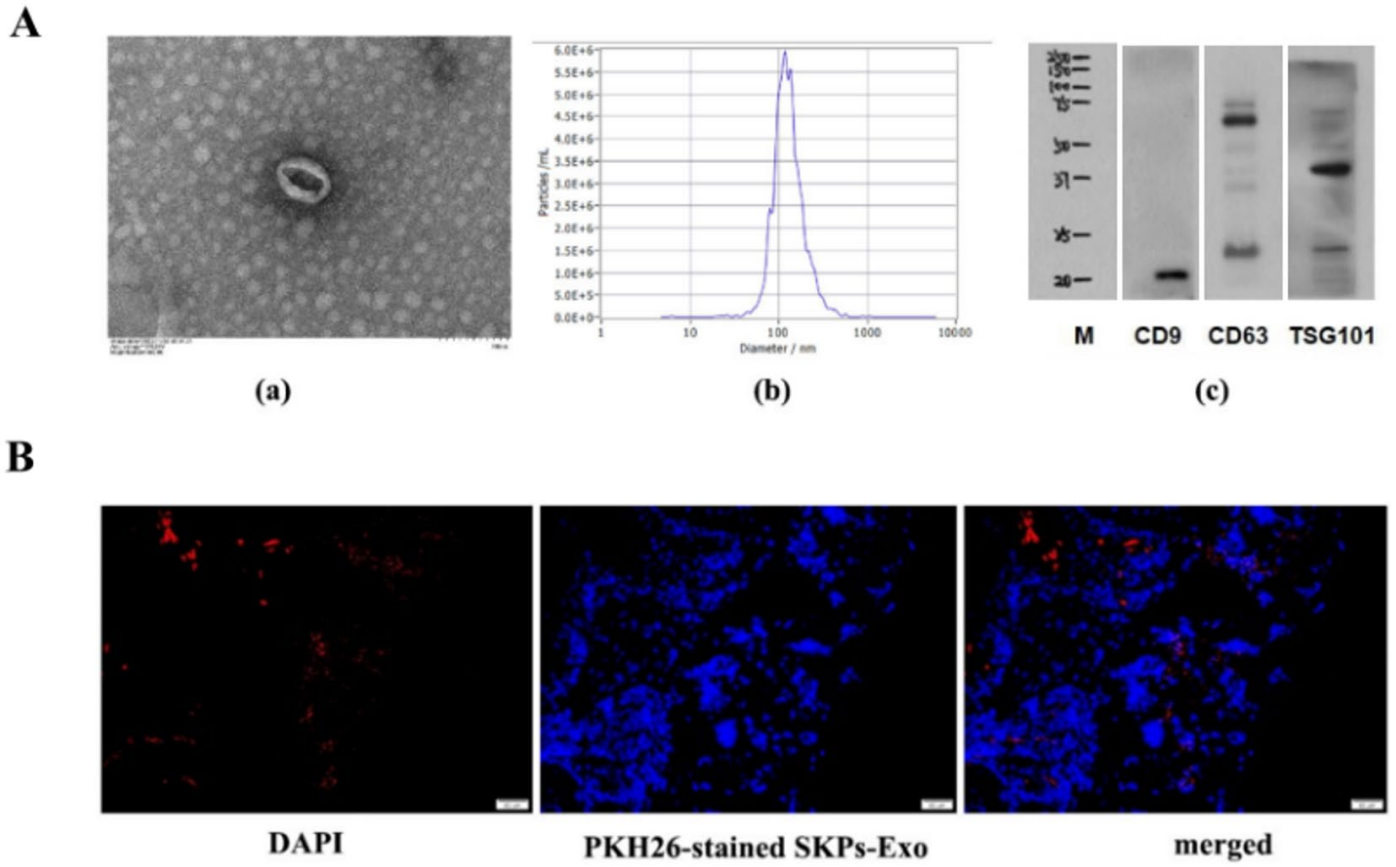
Morphology and feature of SKPs-Exo and condition of SKPs-Exo implantation into the skin of mice under fluorescence microscopy. (**Aa**): Morphology of SKPs-Exo; (**Ab**): Particle size and concentration of SKPs-Exo; (**Ac**): Western blot analysis of exosomal surface markers CD9, CD63, and TSG101 in SKPs-Exo; (**B**): Bcondition of SKPs-Exo implantation into the skin of mice under fluorescence microscopy: Twenty-four hours after implantation, frozen skin sections were prepared and stained with a blue-fluorescent nucleic acid dye (DAPI). SKPs-Exo, exhibiting red-fluorescent staining (PKH26), was observed to diffuse into both the epidermal and dermal layers. (×200)

SKPs-Exo were successfully extracted through ultracentrifugation. Exosomes-derived SKPs exhibited a typical cup-shaped exosome-like structure with an intact morphology under the TEM (Fig. 2Aa). NTA analysis, meanwhile, revealed the average diameter of SKPs-Exo ranging from 30 to 200 nm, which was in line with the expected size of exosomes particles (Fig. 2Ab). By using WB analysis, more importantly, the specific markers of exosomes, i.e., CD9, CD63 and TSG101, remarkably expressed on SKPs-Exo (Fig. 2 c). These confirmed the successful extraction of exosomes from SKPs, laying a foundation for the subsequent research.

### SKPs-Exo alleviated UVB-induced damage in skin appearance

To investigate the effectiveness of SKPs-Exo transplantation into mice, PKH26-labeled SKPs-Exo suspension was injected into mice skin. Subsequent to twenty-four hours, the emergence of red fluorescence in both the epidermis and dermis was discernible under a fluorescence microscope, indicating the successful engraftment of SKPs-Exo in the mice skin (Fig. 2B).

**Fig. 3:**
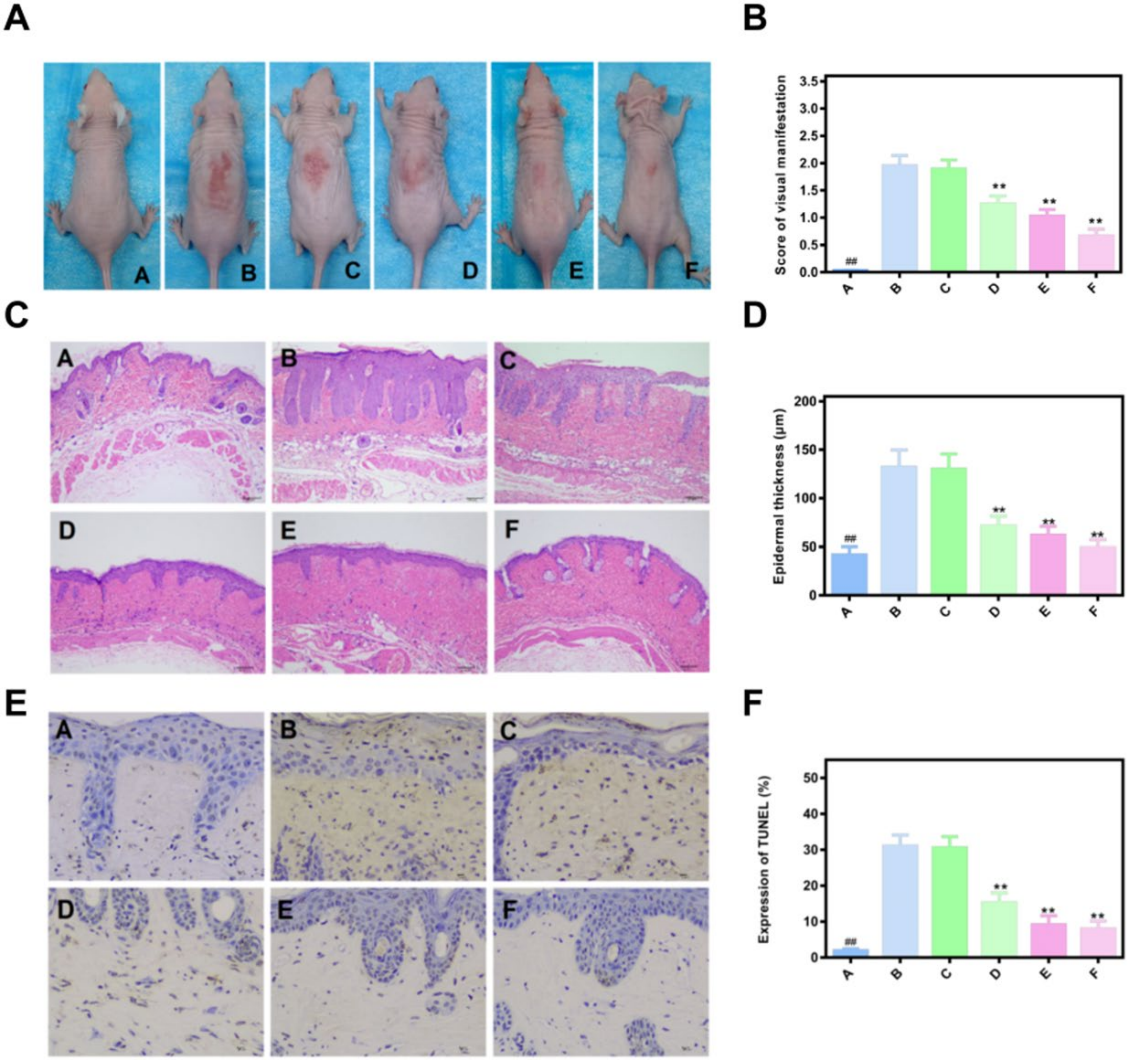
Skin visual manifestations of mice, histopathological alterations of mice’s dorsal skin and apoptotic skin cells of mice by TUNEL staining in each group. (**A**): Visual alterations in the skin of the hairless Balb/C mice in six groups; (**B**): Scores of skin visual manifestation in six groups; (**C**): H&E staining was performed on skin sections derived from six groups (×100); (**D**): The thickness of the epidermis was quantified; (**E**): Immunohistochemical analysis of TUNEL-positive cells expression in six groups; (**F**): Quantitative analysis of TUNEL expression *via* Immunohistochemical analysis in six groups. Compared with the normal group, ## *P* < 0.01; compared with the model group, **P < 0.01. A, Normal group; B, Model group; C, Control group; D, Low-concentration SKPs-Exo group; E, Medium-concentration SKPs-Exo group; F, High-concentration SKPs-Exo group.

After two-week UVB irradiation, photodamage-like eruptions containing edema, erythema and scales, emerged from the dorsal skin of mice in all groups but normal one (Fig. 3A). Nevertheless, these lesions were evidently attenuated by different-concentration SKPs-Exo injection into mice, along with an obvious decrease in scores of skin visual manifestation in three SKPs-Exo treatment groups compared with those in the model group (*P* < 0.01) (Fig. 3A and B). Mice in the control group, conversely, displayed little amelioration with slightly decreased scores but not significantly (*P* > 0.05). Furthermore, all the three SKPs-Exo-treated groups exhibited lower scores than the control group throughout their treatment period, high-concentration group in particular (*P* < 0.01) (Fig. 3A and B).

### SKPs-Exo ameliorated UVB-induced skin histopathology

A histopathological assessment was carried out on the mice exposed to UVB. By H & E staining, the representative histopathological changes appeared in the model group, consisting of thickened epidermis with hyperkeratosis/parakeratosis, acanthosis, spongiosis, as well papillary edema and inflammatory cells infiltration in the dermis (Fig. 3C). However, obvious mitigation of above histopathological alterations, notably epidermal thickness reduction, emerged from different-concentration SKPs-Exo groups (the high-concentration group in special) (*P* < 0.01), instead of the control group (Fig. 3C and D).

### SKPs-Exo mitigated UVB-induced cutaneous apoptosis

Although TUNEL-positive cells rarely appeared in the normal group, UV irradiation multiply the number of TUNEL-positive cells with brownish-yellow condensed nuclei in the model group (Fig. 3E). This phenomenon, however, was reversed by SKPs-Exo treatment. Compared with the model group, TUNEL-positive cells greatly went down in the groups treated with different-concentration SKPs-Exo, especially medium- and high-concentration ones (*P* < 0.01). Alterations in the control group were similar to the model one, nearly barren of reduction in TUNEL-positive cells (*P* > 0.05). When the TUNEL-positive cells in different concentrations of groups were in comparison with each other, significant differences existed in low-concentration group and medium- or high-concentration one (*P* < 0.05) (Fig. 3F).

### SKPs-Exo modulated UVB-induced oxidative/inflammatory indicators

Subsequent to two-week UVB exposure, levels of OS/inflammation-related factors in skin tissue, including ROS, MDA, BACH1, IL-1β, IL-6 and TNF-α, apparently went up in the model group (*p* < 0.05), and those of Nrf2, HO-1, GSH and SOD dramatically went down (*p* < 0.05) (Table 1). Nevertheless, SKPs-Exo, especially the medium and high ones, managed to restore above alterations; namely, medium and high-concentration SKPs-Exo notably lowered the expression of ROS, MDA, BACH1, IL-1β, IL-6 and TNF-α (*p* < 0.05), but elevated the activities of Nrf2, HO-1, GSH and SOD (*P* < 0.05) (Table 1). Despite slight amelioration appearing in low-concentration SKPs-Exo group, it failed to reach statistical significance in comparison with the model group. Similarly, the control group treated with PBS showed few improvements in these parameters, with no significant difference compared with the model group (*P* > 0.05). Relative to the control group, however, two groups treated with medium/high concentration of SKPs-Exo had statistical significance (*P* < 0.05) (Table 1), no difference existing between the medium-concentration group and high-concentration group.

**Table 1:** Effect of SKPs-Exo on skin tissue OS/inflammation-related factors

Parameter		Normal group	Model group	Control group	LC SKP-Exo group	MC SKP-Exo group	HC SKP-Exo group
Nrf2 (ng/mL)		5.56 ± 1.39	3.29 ± 1.21^*^	3.12 ± 1.25	3.76 ± 1.36	5.06 ± 1.32^†#^	5.39 ± 1.29^†#^
HO-1 (mmol/L)		25.19 ± 6.92	12.13 ± 5.33^*^	12.25 ± 5.52	13.26 ± 6.23	22.33 ± 6.65^†#^	23.96 ± 6.71^†#^
GSH (µmol/L)		395.35 ± 36.29	121.67 ± 28.12^*^	119.95 ± 28.19	127.55 ± 31.33	351.55 ± 33.75^†#^	376.52 ± 35.16^†#^
SOD (U/mL)		139.53 ± 22.09	69.62 ± 16.33^*^	68.87 ± 17.15	76.56 ± 21.22	111.55 ± 21.37^†#^	129.58 ± 21.69^†#^
BACH1 (pg/mL)		126.32 ± 21.57	265.76 ± 31.25^*^	262.65 ± 31.36	259.67 ± 35.51	183.65 ± 28.71^†#^	151.07 ± 23.59^†#^
ROS (U/µL)		8.89 ± 3.69	19.26 ± 5.83^*^	18.97 ± 5.91	17.99 ± 5.87	12.65 ± 5.35^†#^	10.06 ± 4.61^†#^
MDA (µmol/L)		3.97 ± 1.39	7.35 ± 1.93^*^	7.31 ± 1.89	6.99 ± 2.05	5.01 ± 1.51^†#^	4.25 ± 1.33^†#^
IL-1β(ng/L)		32.19 ± 9.15	59.35 ± 16.27^*^	58.85 ± 16.35	57.67 ± 17.15	43.25 ± 15.65^†#^	36.17 ± 15.32^†#^
IL-6 (pg/mL)		19.33 ± 8.76	42.99 ± 17.38^*^	42.65 ± 17.32	41.97 ± 16.67	29.09 ± 11.29^†#^	25.26 ± 9.55^†#^
TNF-α (ng/L)		125.56 ± 23.16	237.68 ± 30.36^*^	229.89 ± 31.55	201.32 ± 28.26	171.31 ± 26.22^†#^	135.62 ± 22.71^†#^

* *P* < 0.05, compared to the normal group; † *P* < 0.05, compared to the model group; # *P* < 0.05, compared to the control group.

LC SKP-Exo, Low-concentration SKPs-Exo; MC SKP-Exo, Medium-concentration SKPs-Exo; HC SKP-Exo, High-concentration SKPs-Exo

### SKPs-Exo upregulated Nrf2 and HO-1 expression and downregulated BACH1 and NF-κB expression in UVB-induced photodamage-like mice model

**Fig. 4:**
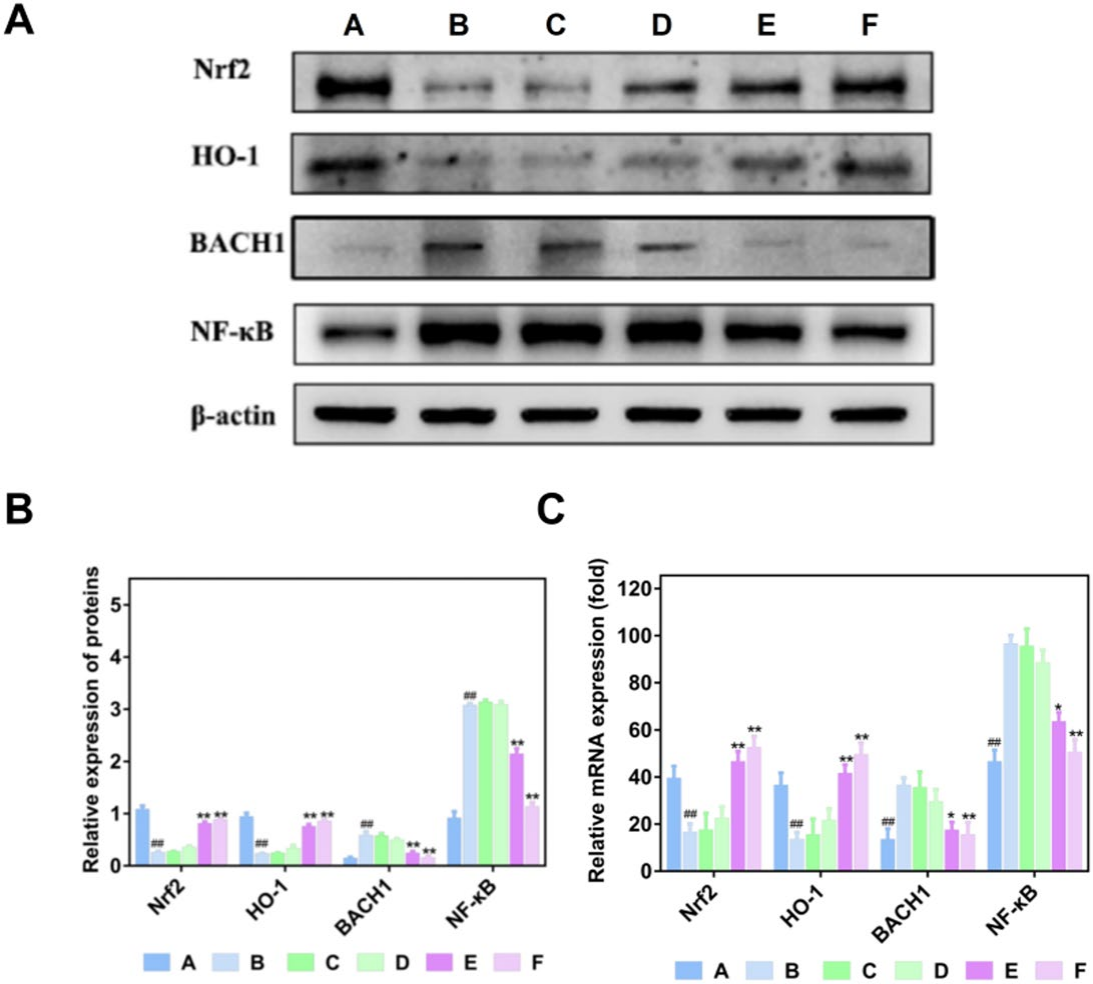
Western blot analysis for the effect of SKPs-Exo on Nrf2, HO-1, BACH1, and NF-κB expression in UVB-induced mice and relative expression levels of Nrf2, Ho-1, Bach1 and Nfkb mRNA in different groups after SKP-Exo intervention. (**A**): Quantitative analysis of protein expression *via* western blot band intensity in six groups; (**B**): Relative expression levels of Nrf2, HO-1, BACH1 and NF-κB in six groups; (**C**): Relative expression levels of Nrf2, Ho-1, Bach1 and Nfkb mRNA in different groups after SKP-Exo intervention. Compared with the normal group, ## *P* < 0.01; compared with the model group, **P < 0.01. A, Normal group; B, Model group; C, Control group; (**D**), Low-concentration SKPs-Exo group; E, Medium-concentration SKPs-Exo group; F, High-concentration SKPs-Exo group.

UVB irradiation remarkably decreased the expression of Nrf2 and HO-1 proteins in the model group mice (*p* < 0.01), while increased that of BACH1 and NF-κB ones (*p* < 0.01) (Fig. 4A and B). Two weeks post SKPs-Exo treatment, nevertheless, the Nrf2 and HO-1 proteins in the medium- and high-concentration SKPs-Exo groups apparently ascended (*p* < 0.01), along with a descent in the proteins of BACH1 and NF-κB (*p* < 0.01) (Fig. 4A and B). Although the low-concentration SKPs-Exo group exhibited slight improvements in these proteins, few significant differences existed compared with the model group (*p* > 0.05). In addition, similar alterations occurred in the control group to the low-concentration SKPs-Exo group or model one, difference rarely appearing among each other (*p* > 0.05).

### SKPs-Exo enhanced the mRNA levels of *Nrf2* and Ho-1 and lowered those of Bach1 and *Nf-κb* in UVB-induced photodamage-like mice model

In comparison with the normal group, the model group after UVB irradiation exhibited the reduced expression of *Nrf2* and *Ho-1* mRNA (*p* < 0.01), and the enhanced expression of *Bach1* and *Nfkb* mRNA (*p* < 0.01). Conversely, injection of SKPs-Exo, mainly in medium and high concentration, give rise to higher mRNA levels of *Nrf2* and *Ho-1* and lower mRNA levels of *Bach1* and *Nfkb* (*p* < 0.05); whereas low-concentration SKPs-Exo group experienced slight changes in these parameters, but no difference appeared relative to both the control group and the model one. Similar alterations also occurred in the control group, with little statistical significance observed when compared to the model group (*P* > 0.05) (Fig. 4C).

### Visual and histological manifestations of 3D skin equivalents

**Fig. 5:**
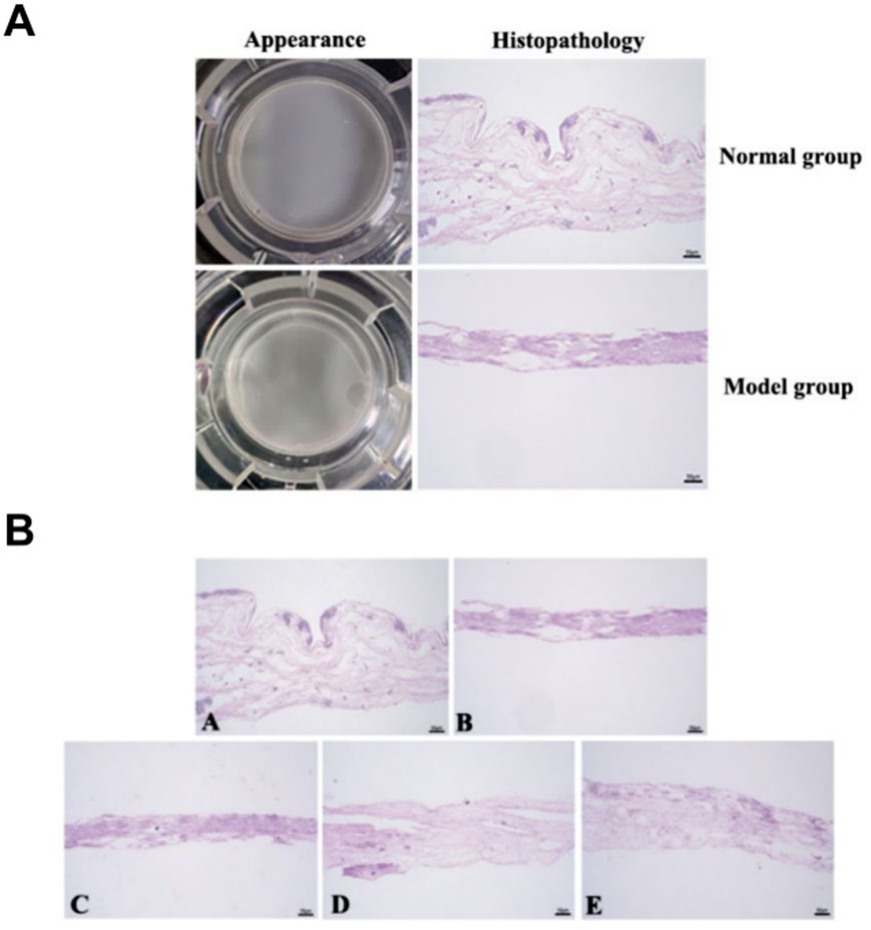
Appearance and histopathology of 3D skin models and histopathology of 3D skin model in each group. (**A**): Appearance and histopathology of 3D skin models; (**B**): Histopathology of 3D skin model in each group. A, normal group; B, model group; C, control group; D, medium-concentration group; E, high-concentration group.

Same as the descriptions in our previous reports, the normal 3D skin in appearance presented as an elastic, tear-resistant, milky-white membrane-like structure; histologically, it displayed a distinct layered structure that KCs arranged tightly in epidermis and reddish collagen fibers interwove with spindle-shaped FBs in dermis (Fig. 5A).

After exposure to UVB, nevertheless, 3D skin appeared weaker, shrunken and wrinkled at the edges. In histology, the epidermis became thinning, in which KCs arranged irregularly and sparsely with cellular swollen/ necrosis and nuclei condensation, whereas the dermis underwent collagen degradation and FBs reduction (Fig. 5A).

### SKPs-Exo ameliorated the histological structure of photodamaged-like 3D skin

Following SKPs-Exo implantation into 3D skin, both medium-concentration and high-concentration groups exhibited neatly aligned, clearly defined cell layers in epidermis compared with the model group or control group, in which nuclei remained full without shrinkage; although FBs slightly reduced, scarce cellular necrosis or collagen degradation emerged from the dermis and the overall structure remained full closer to that of the normal group (Fig. 5B).

### SKPs-Exo inhibited OS/ inflammation in photodamaged 3D skin

**Fig. 6:**
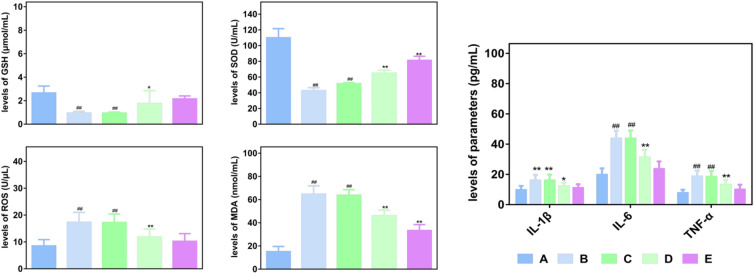
Inflammatory/oxidative parameters of 3D skin model in each group. Compared with the normal group, ## *P* < 0.01; compared with the model group, *P < 0.05, **P < 0.01. A, normal group; B, model group; C, control group; D, medium-concentration group; E, high-concentration group.

Compared with the normal group, UVB significantly enhanced levels of ROS, MDA, IL-1β, IL-6 and TNF-α, while lowering those of SOD and GSH in the model group (*P* < 0.01). SKPs-Exo at medium or high concentrations, however, reversed these changes (*P* < 0.05); instead, PBS failed to work on inflammatory /OS indicators in the control group (*P* > 0.05) (Fig. 6).

### SKPs-Exo regulated the expression of proteins in photo-damaged 3D skin

**Fig. 7:**
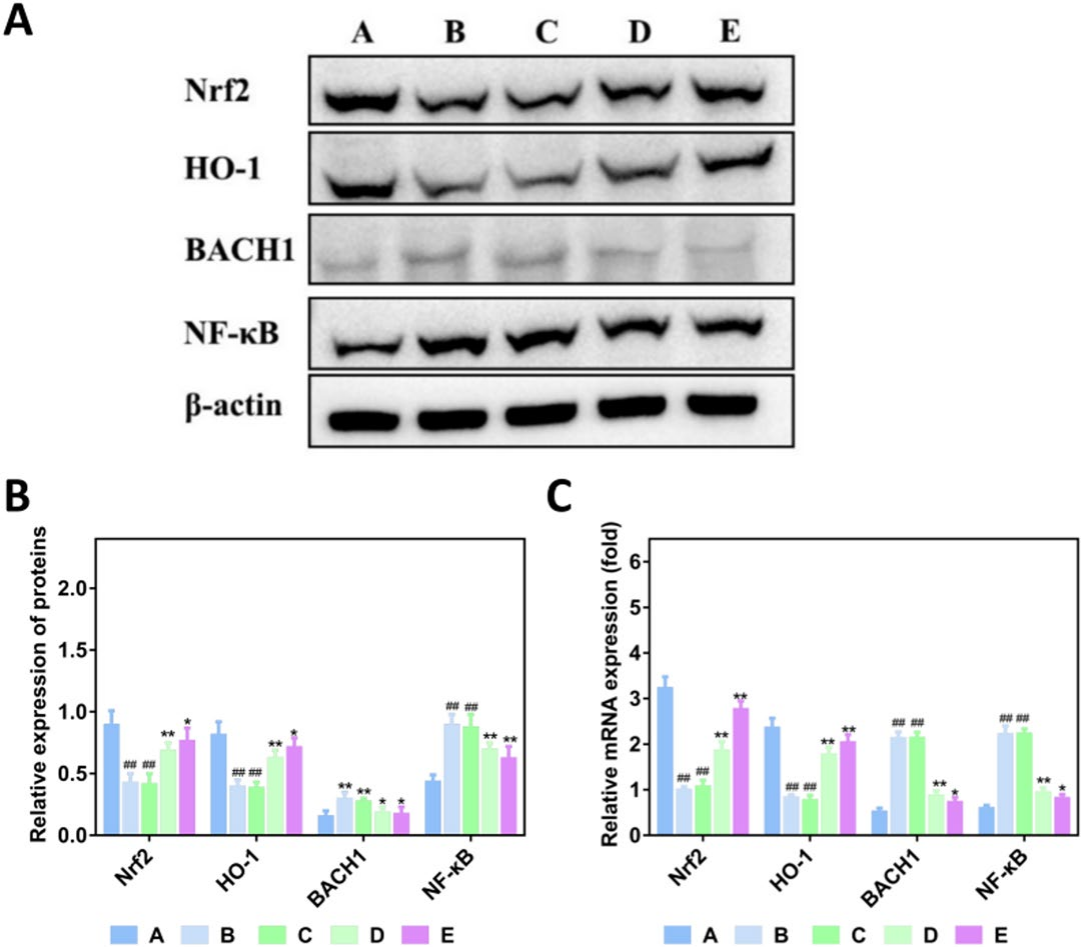
Expression of different proteins and genes after SKPs-Exo intervention in 3D skin model. (**A**-**B**): Expression of different proteins after SKPs-Exo intervention in 3D skin model; (**C**): Expression of different genes after SKPs-Exo intervention in 3D skin model. A, normal group; B, model group; C, control group; D, medium-concentration group; E, high-concentration group.

After UVB irradiation, Nrf2 and HO-1 expression in the 3D skin model fell markedly while BACH1 and NF-κB greatly rose (*P* < 0.01). On the contrary, these parameters suffered a total reversal under the presence of SKPs-Exo; namely, medium- or high-concentration SKPs-Exo restored the expression of Nrf2 and HO-1, and suppressed that of BACH1 and NF-κB, with the high concentration showing the strongest effect (*P* < 0.01 vs. the model group). PBS alone produced no change in the control group compared to the model group (*P* > 0.05). (shown in Fig. 7A-B)

### SKPs-Exo orchestrated the expression of genes in photo-damaged 3D skin

In the 3D skin model, UV exposure significantly downregulated *Nrf2* and *HO-1* mRNA levels, concomitantly upregulating *BACH1* and *NF-κB* (*P* < 0.01). Nevertheless, treatment with SKPs-Exo at either medium or high concentrations led to a significant increase in the mRNA expression of *Nrf2* and *HO-1* (*P* < 0.01), accompanied by a decrease in *BACH1* level (*P* < 0.05); *NF-κB* mRNA levels were markedly reduced, but only under the high-concentration condition (*P* < 0.01), instead of the medium one (*P* > 0.05). Besides, PBS treatment rarely alter the expression of any transcripts in the control group compared to the model one (*P* > 0.05) (Fig. 7C).

## Discussion

The present study uncovers a new effect of SKPs-Exo on a murine model of skin photodamage *in vivo and in vitro*. First, SKPs-Exo were successfully extracted from the supernatant of SKPs through a high-speed ultracentrifugation. Then, these exosomes at different concentrations were respectively injected into the well-constructed skin photodamage-like mouse models. Subsequently, skin symptoms, visual scoring and histopathological manifestations were relieved by an increasing-concentration exosomal treatment, the highest one in particular. Besides, SKPs-Exo not only reduced cutaneous apoptosis, but also suppressed the production of inflammatory and oxidative mediators. Lastly, levels of Nrf2 and HO-1, in the presence of SKPs-Exo, ramped up and those of BACH1 and NF-κB dropped down. Meanwhile, these results had also been validated in photodamaged-like 3D skin model. We found that SKPs-Exo could restore epidermal structure, reduce dermal necrosis and reverse UVB-induced OS/inflammation in photodamaged-like 3D skin model. Mechanistically, it could regulate Nrf2/HO-1 (antioxidant) and BACH1/NF-κB (pro-inflammatory) at protein/mRNA levels, with high concentration exerting stronger effects. These findings throw light on the capacity of SKPs-Exo in mitigating inflammation and OS *via* activation of the Nrf2/HO-1 pathway and inactivation of the BACH1/NF-κB signals to mitigate experimental skin photodamage, further to enlighten the exosomal mechanism of SKPs treating skin photodamage.

Currently, exosomes therapy, stem cell-derived ones in especial, is emerging as an innovative approach to address injuries and diseases^25–28^. In our previous studies, we demonstrated the powerful capacity of SKPs on skin photodamage, possibly relating to the exosomal pathway^17,18^. So, to clarify it, we extracted exosome-shaped vesicles from SKPs and identified them as SKPs-Exo, reliant on the fact that the vesicles exhibit a typical cup-shaped bilayer membrane structure with 30–200 nm size and present the specific markers of exosomes like CD9, CD63 and TSG. The characteristics of above vesicles coincided with those of exosomes reported in documents from Tan and Doyle et al.^29,30^. Thus, it would be confirmed that SKPs-Exo have successfully extracted from SKPs, the findings providing exosomes for the subsequent studies.

To investigate the effect of SKPs-Exo against skin photodamage, we implanted them into the skin of UVB-induced mice at different concentrations, i.e. low concentration, medium concentration and high concentration. As expected, SKPs-Exo injection, whether low or high concentration, remarkably alleviated the visual appearance of UVB-damaged mice and greatly ameliorated histopathological alterations, effectively reducing skin apoptosis. Conversely, mice in the control group seldom exhibited amelioration, as evidenced by both visual assessment and histopathological examination. These outcomes underscore the significant role of SKPs-Exo in mitigating and managing skin photodamage, aligning with the findings of prior research. Reports from Wang and Shen also revealed that the implantation of stem cells-derived exosomes diminished OS-induced skin damage via regulating Nrf2 signal^32,33^. Our study, thus, indicated that SKPs-Exo could indeed alleviate the histopathological damage and apoptosis of mice skin under UV irradiation.

Given the efficacy of SKPs-Exo in controlling skin photodamage, it is imperative to elucidate their therapeutic mechanisms further. Prolonged or high-dose UV irradiation boosts ROS accumulation in the skin, thereby inducing BACH1/Mafs heterodimers, inactivating Nrf2 and escalating OS in cutaneous cells, consequently culminating in the occurrence of skin photodamage^33,34^. In this process, Nrf2 and BACH1, key players from the Cap-N-Collar family, exert crucial influences on the ARE-driven HO-1 expression in response to OS^35–37^. Excessive ROS generation from UV promote BACH1 upregulation and Nrf2 ubiquitination, leading to Nrf2 inactivation and thereby Nrf2/ARE pathway retardant. Consequently, the presence of BACH1 and the inactivation of Nrf2 facilitate an apparent reduction in antioxidant enzymes/proteins, HO-1 in particular, followed by the enhancement of inflammatory indicators (e.g. NF-κB, IL-1β, IL-6 and TNF-α), thus exacerbating OS-induced damage and resulting in photodamage^5,9,10,33–37^. The alterations of these biomarkers echoed in our present study. Indeed, BACH1 and NF-κB significantly went up, whereas Nrf2 and HO-1 went down after UV irradiation, subsequently with the decreased activities of SOD and GSH, and the increased levels of ROS, MDA, IL-1β, IL-6 and TNF-α. As the transcriptional repressor of HO-1, BACH1 presence undercuts the ability of Nrf2 to activate HO-1^7^; while the inactivation of Nrf2 and HO-1 in turn exaggerates photooxidative damage and inflammation in mice skin^38–40^, demonstrating the vital roles of BACH1 and Nrf2/HO-1 in skin photodamage. Collectively, these results suggest that BACH1 upregulation and Nrf2 inactivation, triggered by excessive or high-dose UV, are crucial players in the exacerbation of OS and the initiation of skin photodamage.

Our findings, nevertheless, revealed that SKPs-Exo has the ability to reverse this process. We in the current study discovered that SKPs-Exo injection, except for low- concentration one, notably reduced the levels of ROS, MDA, NF-κB, IL-1β, IL-6 and TNF-α, while enhanced the activities of GSH and SOD in UVB-damaged skin, further suggesting that medium/high-concentration SKPs-Exo could effectively mitigate OS / inflammation and strengthen antioxidant defense. What’s more important, Nrf2 and HO-1 exhibited strong expression in the presence of medium/high-concentration SKPs-Exo, whereas BACH1 weakly expressed in the UVB-damaged skin of mice; conversely, PBS rarely worked in these mice, the same as SKPs-Exo in low concentration. Although the levels of OS-and inflammation-related parameters in the high-concentration group differed from those in the medium-concentration one, few significant differences existed between them. In addition, we had successfully constructed a photodamaged-like 3D skin model, in which we observed phenomena similar to the experimental results mentioned above. Our findings suggest that SKPs-Exo have the ability to restrain BACH1 and excite Nrf2/HO-1, further signifying the protective effect of SKPs-Exo against skin photodamage in connection with the mitigation of OS and inflammation *via* inactivation of BACH1 and activation of Nrf2/HO-1 signaling pathway. Really, Nrf2 activation is pivotal for cells/tissues in their defense against OS, which facilitates HO-1 upregulation, especially in the case of BACH1 inactivation, further response to oxidative challenges. Hence, the Nrf2/HO-1 pathway activation effectively alleviates UV-triggered cytotoxicity and apoptosis in skin. Likewise, numerous documents have extensively revealed the protective role of exosomes against OS^31,32,41–45^. Findings from recent studies confirm that the primary mechanism of exosomes combating OS centers around the activation of Nrf2 that competitively recognize the same site to inhibit BACH1^31,32,41–45^. These studies have uncovered that elevation of Nrf2 overexpression in exosomes can shield cells from OS-induced apoptosis or cytotoxicity, while also stimulating the activity of HO-1 and SOD. Altogether, above findings underscore the protective role of exosomes in combating OS, with Nrf2 being pivotal in orchestrating anti-oxidative stress, anti-inflammation and anti-photodermatosis. Consequently, Nrf2/HO-1 activation along with BACH1 in stillness emerges as the crucial target for SKPs-Exo safeguarding cells and tissues against skin photodamage, which aligns with our prior outcomes^17,18^. Thus, our present study not only clarify the specific mechanism of SKPs in skin photodamage involving SKPs-derived exosome pathway, but also enlighten the therapeutic mechanism of SKPs-Exo treating skin photodamage primarily linking to the high expression of Nrf2/HO-1 and the inhibition of BACH1.

## Materials and methods

### Mice and human samples

Six-week-old male hairless Balb/C mice and 1-3-day-old neonatal ones (Chengdu Dashuo Experimental Animals Co., Ltd. in Chengdu, China) were housed at 23 ± 3 °C temperature and 55 ± 5% humidity under a standard 12 h light- dark cycle, with food and water *ad libitum*. Animal experiments were performed in the SPF animal laboratory at the Center of Experimental Animal of Southwest Medical University. Meanwhile, human foreskin samples were collected, with parental consent, from 3-5-year-old patients undergoing routine circumcision.

### Ethics statement

All experiments involving human tissue samples were carried out in accordance with the Declaration of Helsinki and relevant national/international guidelines and regulations. The study was approved by the Human Ethics Committee of the Affiliated Hospital of Southwest Medical University (Permit Number: KY2024453). Human foreskin samples were collected from 3–5-year-old patients undergoing routine circumcision, and written informed consent was obtained from their parents or legal guardians prior to sample collection.

Animal experiments were performed in accordance with the Guide for the Care and Use of Laboratory Animals and approved by the Animal Experimental Ethics Management Committee of Southwest Medical University (Permit Number: 20230065).

### Extraction and identification of exosomes

As our previously described methods, SKPs were isolated from the dermal skin of neonatal mice and cultured in serum-free SKPs medium (3: 1 DMEM/F12 comprising 2% B27 supplement, 40 ng/mL FGF2, 20 ng/mL EGF and 0.1% P/S; Invitrogen, Carlsbad, CA, USA)^17,18^.

For the extraction of SKPs-Exo, the supernatant from SKPs was initially collected after sequential centrifugation at 2000 g and 12,000 g for 10 min each. Following this, the supernatant was filtered through a 0.22 µ m membrane and subjected to further centrifugation at 4 ℃,120,000 g for 90 min. Subsequent to the aspiration of supernatant, the precipitate was resuspended with PBS and centrifugated again at 120,000 g for 70 min. Lastly, exosomes were successfully extracted and stored at -80 ° C for subsequent applications.

For the identification of SKPs-Exo, transmission electron microscopy (TEM) and nanoparticle tracking analysis (NTA) were introduced to assess the morphology, size, distribution, diameter and concentration of exosomes, while western blotting (WB) to analyze the specific markers of SKPs-Exo, like CD9, CD63, TSG101, with GeneChem’s technical backing (Shanghai, China).

### Label of SKPs-Exo with PKH26 fluorescence

Following the manufacturer’s guidelines, 100 µL of 250-fold diluted PKH26 solution was added to the SKPs-Exo suspension. After a 20-minute incubation at 4 °C, the exosomes were labeled with red fluorescence. The PKH26-labeled exosome suspension was then prepared for injection into mice or 3D skin models, and subsequent tracking via fluorescence microscopy.

### Animal experiments

#### Group division

Thirty-six hairless BALB/c mice were randomly divided into the following six groups (*n* = 6): normal group, model group, control group, low-concentration SKPs-Exo group, medium-concentration SKPs-Exo group and high-concentration SKPs-Exo group.

#### Preparation of different-concentration SKPs-Exo suspension

By BCA protein concentration assay, the concentration of SKPs-Exo suspension was determined as about 200 µg/mL. SKPs-Exo suspension at this concentration then was respectively diluted into high, medium and low concentrations with different-volume PBS, namely, high-concentration SKP-Exo suspension at 100 µg/mL, medium-concentration SKP-Exo suspension at 50 µg/mL and low-concentration SKP-Exo suspension at 25 µg/mL.

#### Injection of SKPs-Exo into skin photodamage-like mice

Mice in all groups but the normal, every other day, dorsally suffered a two-week UVB exposure (180 mJ/cm^2^) with a lighting distance of 20 cm. After that, mice in the SKPs-Exo groups received a subcutaneous injection of SKPs-Exo suspension on the back at doses of 100 µL per mouse, corresponding to their respective concentrations, twice weekly for an additional two weeks, while UVB irradiation continued as before. The control group in the same way was given an equal-volume PBS. The model group got solely UVB irradiation and the normal one received nothing. Over this time, skin lesions were monitored daily and photographs were taken document the progression. After the experiments, mice were humanely euthanized and samples of serum and dorsal skin were collected. These specimens then were subjected to histopathological observation and oxidative/inflammatory indicators measurement.

### Experiments in vitro

#### Reconstruction of 3D skin model

To establish the 3D skin model, fibroblasts (FBs) and keratinocytes (KCs) were firstly isolated from the dermal and epidermal layers of foreskin, respectively, and cultured following our established protocols^18^. Subsequently, passage-2-3 FBs were mixed with type I rat-tail collagen; after 3–4 days of immersion in the serum-free keratinocyte-SFM, the collagen gels served as a dermal equivalent; KCs were then inoculated on the surface of the gels in a Transwell insert, fed with serum-free keratinocyte-SFM for 3 days, and raised to the air-liquid interface for a further 7 days to generate a normal 3D skin model, exactly as we described previously^18^.

#### Grouping

The reconstructed 3D skin models were randomly assigned to the following five groups: normal group (no intervention), model group (UVB irradiation), control group (PBS + UVB), medium-concentration group (medium-concentration SKPs-Exo + UVB) and high-concentration group (high-concentration SKPs-Exo + UVB). Twenty-four hours post UVB irradiation, all groups were examined.

#### Preparation of SKPs-Exo suspension

Same as the method in part 2.5.2, different-concentration SKPs-Exo suspension was prepared with PBS at high (100 µg/mL) and medium (50 µg/mL) concentrations.

#### SKPs-Exo injection into 3D skin models

Twenty-four hours prior to UV irradiation, PBS, 50 µg/mL of SKPs-Exo suspension and 100 µg/mL of SKPs-Exo suspension were separately injected into the control group, medium-concentration group and high-concentration group. About 100 µL of SKPs-Exo suspension or PBS was implanted at multiple points into 3D skin by using a gauge needle. The models were subsequently cultured at the air-liquid interface for 24 h, with dermal side down in the medium.

#### UVB irradiation

Twenty-four hours post SKP-Exo injection, the above 3D skin models were rinsed twice with PBS and exposed to a solar simulator (SUV1000, 290–400 nm, Shanghai sigma High Tech Co Ltd., Shanghai, China); all groups except the normal one received a single 60-s exposure (UVB 90 mJ/cm²) delivered from 20 cm away with the lid removed. After that, the 3D skin models were returned to the air-liquid interface for a further 24-hour-culture. Finally, both the supernatants and 3D skin were harvested for follow-up experiments.

### Visual skin evaluation

The visual alterations in the mice’s skin were evaluated by employing an adapted scoring system of skin photodamage specifically tailored for the experiment. This system was refined based on our previous adjustment to the established criteria for assessing skin photodamage^18^. (shown in Supplementary Table 1)

### Preparation of skin tissue for histopathological examination

Mice dorsal skin or 3D skin specimens were sequentially fixed with 4% paraformaldehyde solution, embedded in paraffin, and sectioned into 3-µm-thick slices for Hematoxylin and Eosin (H&E) staining and TUNEL staining. Epidermal thickness was measured in samples stained with H&E, using standard histological procedures for H&E staining. The epidermal thickness, spanning from the granular layer to the basal layer, was assessed at three randomly selected sites on each tissue section. For each site, two distinct fields were captured at high resolution under the microscope. The mean values (± SD) of epidermal thickness were determined independently by two investigators.

### TUNEL staining for skin cells apoptosis

Apoptotic cells were quantified via TUNEL staining with a kit from abcam, UK, following the manufacturer’s instructions. The process involved deparaffinizing and rehydrating tissue sections, performing antigen retrieval, and then conducting TUNEL staining as directed. Positive cells were analyzed using microscopy.

### Determination of OS/inflammation-related indicators in skin tissue

As the method previously described by us, fresh samples of mice skin and 3D skin tissue were homogenized in cold PBS (1:4 w/v) using a Polytron homogenizer for five minutes on ice. The homogenate was then subjected to centrifugation at 4000 r.p.m. 4 °C for 15 min to yield the cytosolic fraction.

For the measurement of OS/inflammation-related markers containing ROS, MDA, BACH1, Nrf2, HO-1, GSH, SOD, IL-1β, IL-6 and TNF-α in mice skin or 3D skin tissue, kits of enzyme-linked immunosorbent assay (ELISA) and colorimetric method came from Nanjing Jiancheng Technology Co., Ltd (Nanjing, China). ELISA was available for ROS, BACH1, Nrf2, HO-1, IL-1β, IL-6 and TNF-α, while colorimetric method for MDA, GSH and SOD. The assays were carried out as the manufacturer’s guidelines for the kits, and optical density (OD) values of these markers were detected under Microplate Reader (Thermo Fisher Scientific, USA).

### Western blotting (WB) analysis for Nrf2, HO-1, BACH1 and NF-κB

For the detection of Nrf2, HO-1, BACH1 and NF-κB proteins, WB analysis was introduced. As previously described, mice skin or 3D skin samples were minced and subsequently processed with lysate and protein buffers to facilitate total proteins extraction. Following measurement of concentration by BCA assay, proteins were resolved by SDS-PAGE, then transferred to PVDF membranes. After blocking with 5% milk in TBST, membranes were incubated with primary antibodies (HO-1, Nrf2, BACH1, NF-κB, all at 1:1000 dilution) overnight at 4 °C, followed by HRP-conjugated secondary antibody (1:5000). Bands were visualized using an ECL system and quantified with image analysis software.

### Real-time quantitative reverse transcription PCR (qRT-PCR) analysis

To detect the mRNA levels of *Nrf2*, *Ho-1*, *Bach1* and *Nf-κb* genes, total RNA was extracted from mice skin or 3D skin samples by TRIzol RNA extraction kit following the manufacturer’s protocol. All primers used in the experiments were designed and synthesized by Shenggong Bioengineering Technology (Shanghai, China) (shown in Supplementary Tables 2 and 3). The expression of target genes was normalized against that of the reference gene, β-actin, serving as an internal control.

### Statistical analysis

Statistical analysis was conducted using SPSS version 22.0. Data are expressed as mean ± standard deviation (SD). Comparisons between two groups were performed by Student’s t-test, while multiple group comparisons by Least Significant Difference (LSD) analysis following a one-way ANOVA. Statistical significance was defined as a p-value less than 0.05. Data plots was analyzed by Orange 8.5 and GraphPad Prism 8.0.

## Conclusion

This study demonstrates that SKPs-Exo improve UVB-induced photodamage symptoms in both mouse models and 3D skin models by activating the Nrf2/HO-1 antioxidant pathway and suppressing the BACH1/NF-κB pro-inflammatory pathway, providing a potential therapeutic direction for skin photodamage. However, this work has several limitations. First, the key effector molecules in SKPs-Exo that mediate the protective effects remain unidentified, failing to clarify the core bioactive components initiating the regulatory effects on the above signaling pathways.Second, long-term safety evaluations of SKPs-Exo are lacking; the research only observes short-term therapeutic effects within a limited experimental cycle, with no data on potential long-term side effects such as local immune responses or cytotoxicity caused by exosome accumulation. Third, the experimental models have inherent limitations in simulating human skin photodamage; the hairless mouse model has species-specific skin differences, and the 3D skin model lacks complete skin appendages and the native immune microenvironment, leading to a gap between experimental results and clinical manifestations.Fourth, the study only explores the local injection administration route, without verifying the efficacy, skin penetration and stability of more clinically feasible routes such as topical smearing. Fifth, the optimal therapeutic window of SKPs-Exo is not clarified; although medium and high concentrations show better effects, the critical effective concentration and optimal administration frequency are yet to be determined. Future research will focus on identifying key effector molecules, conducting long-term safety assessments, constructing more humanized skin models, exploring diverse administration routes and optimizing the dose regimen, and further verifying the clinical application potential of SKPs-Exo through clinical research.

## Supplementary Information

Below is the link to the electronic supplementary material.

Supplementary Material 1

## Data Availability

The datasets generated during this study will be made publicly available in the scientic report upon acceptance of the manuscript for publication.
