# Supplementary material for: Protective effects of skin-derived precursor cell exosomes against UVB-induced skin photodamage
Source: Sci Rep. 2026 Apr 28;16:19851. doi: 10.1038/s41598-026-48604-1 (PMC13315955; doi:10.1038/s41598-026-48604-1)
Supplement: Supplementary file 1 — Supplementary Material 1 [file 41598_2026_48604_MOESM1_ESM.docx]

**Supplementary Figures**

**Supplementary Table 1 Scoring system for assessing mice skin photodamage**

| Score |  | Skin visual manifestation |
| --- | --- | --- |
| 0 |  | Normal skin with pink, smooth and plump appearance |
| 0.5 |  | Mild erythema appearing on exposed site |
| 1 |  | Moderate erythema, mild edema and few scales on exposed site |
| 1.5 |  | Obvious erythema and scales, severe edema and mild roughness on exposed site |
| 2 |  | Gray skin with lot of scales, obvious roughness, hypertrophy and wrinkles on exposed site |

**Supplementary Table 2 The primers of *Nrf2*, *Ho-1*, *Bach1* and *Nf-κb* genes in mice skin**

| **Genes** | **Forward** | **Reverse** |
| --- | --- | --- |
| *Nrf2* | 5'-TCGAGTCTCCATGCAGCTACGG-3' | 5'-CGGTGGCGATCATCTGTGTCTG-3' |
| *Ho-1* | 5'-TCCTTGTACCATATCTACACGG-3' | 5'-GAGACGCTTTACATAGTGCTGT-3' |
| *Bach1* | 5'- CGGATAATTTCGCTCTCACG -3' | 5'- TAAGGAAAGCGGGCAGTCGG -3' |
| *Nf-κb* | 5'-TCGAGTCTCCATGCAGCTACGG-3' | 5'-CGGTGGCGATCATCTGTGTCTG-3' |
| *β-actin* | 5'-CTACCTCATGAAGATCCTGACC-3' | 5'-CACAGCTTCTCTTTGATGTCAC-3' |

**Supplementary Table 3 The primers of *Nrf2*, *HO-1*, *BACH1* and *NF-κB* genes in 3D skin models**

| **Genes** | **Forward** | **Reverse** |
| --- | --- | --- |
| *Nrf2* | 5'- ACAATGAGGTTTCTTCGGCTACG-3' | 5'- CGTCTAAATCAACAGGGGCTACC-3' |
| *HO-1* | 5'- GCAGGAGGTCATCCCCTACACAC-3' | 5'- TCTGGGCAATCTTTTTGAGCACC -3' |
| *BACH1* | 5'- CACCGAAGGAGACAGTGAATCC -3' | 5'- GCTGTTCTGGAGTAAGCTTGTGC-3' |
| *NF-κB* | 5'- CCAGACCAACAACAACCCCTTCC-3' | 5'- GCAGAGCCGCACAGCATTCAG -3' |
| *β-actin* | 5'- TGGCACCCAGCACAATGAA -3' | 5'- GAAGCATTTGCGGTGGACG -3' |

**Supplementary Table 4 List of Abbreviations**

| **Abbreviation** | **Full Name** |
| --- | --- |
| SKPs | Skin-derived Precursor Cells |
| Exo | Exosomes |
| SKPs-Exo | SKPs-derived Exosomes |
| OS | Oxidative Stress |
| ROS | Reactive Oxygen Species |
| MDA | Malondialdehyde |
| GSH | Glutathione |
| SOD | Superoxide Dismutase |
| IL-1β | Interleukin-1β |
| IL-6 | Interleukin-6 |
| TNF-α | Tumor Necrosis Factor-α |
| NF-κB | Nuclear Factor-kappa B |
| HO-1 | Heme Oxygenase-1 |
| TEM | Transmission Electron Microscopy |
| NTA | Nanoparticle Tracking Analysis |
| WB | Western Blotting |
| PBS | Phosphate-Buffered Saline |
| TUNEL | Terminal Deoxynucleotidyl Transferase dUTP Nick-End Labeling |
| ELISA | Enzyme-Linked Immunosorbent Assay |
| qRT-PCR | Real-time Quantitative Reverse Transcription Polymerase Chain Reaction |
| FBs | Fibroblasts |
| KCs | Keratinocytes |
| DMEM/F12 | Dulbecco's Modified Eagle Medium/Nutrient Mixture F-12 |
| B27 | B27 Supplement |
| FGF2 | Fibroblast Growth Factor 2 |
| EGF | Epidermal Growth Factor |
| P/S | Penicillin/Streptomycin |
| BCA | Bicinchoninic Acid |
| SDS-PAGE | Sodium Dodecyl Sulfate-Polyacrylamide Gel Electrophoresis |
| PVDF | Polyvinylidene Fluoride |
| TBST | Tris-Buffered Saline with Tween 20 |
| HRP | Horseradish Peroxidase |
| ECL | Enhanced Chemiluminescence |
| ANOVA | Analysis of Variance |
| LSD | Least Significant Difference |
| SPF | Specific Pathogen-Free |
| OD | Optical Density |


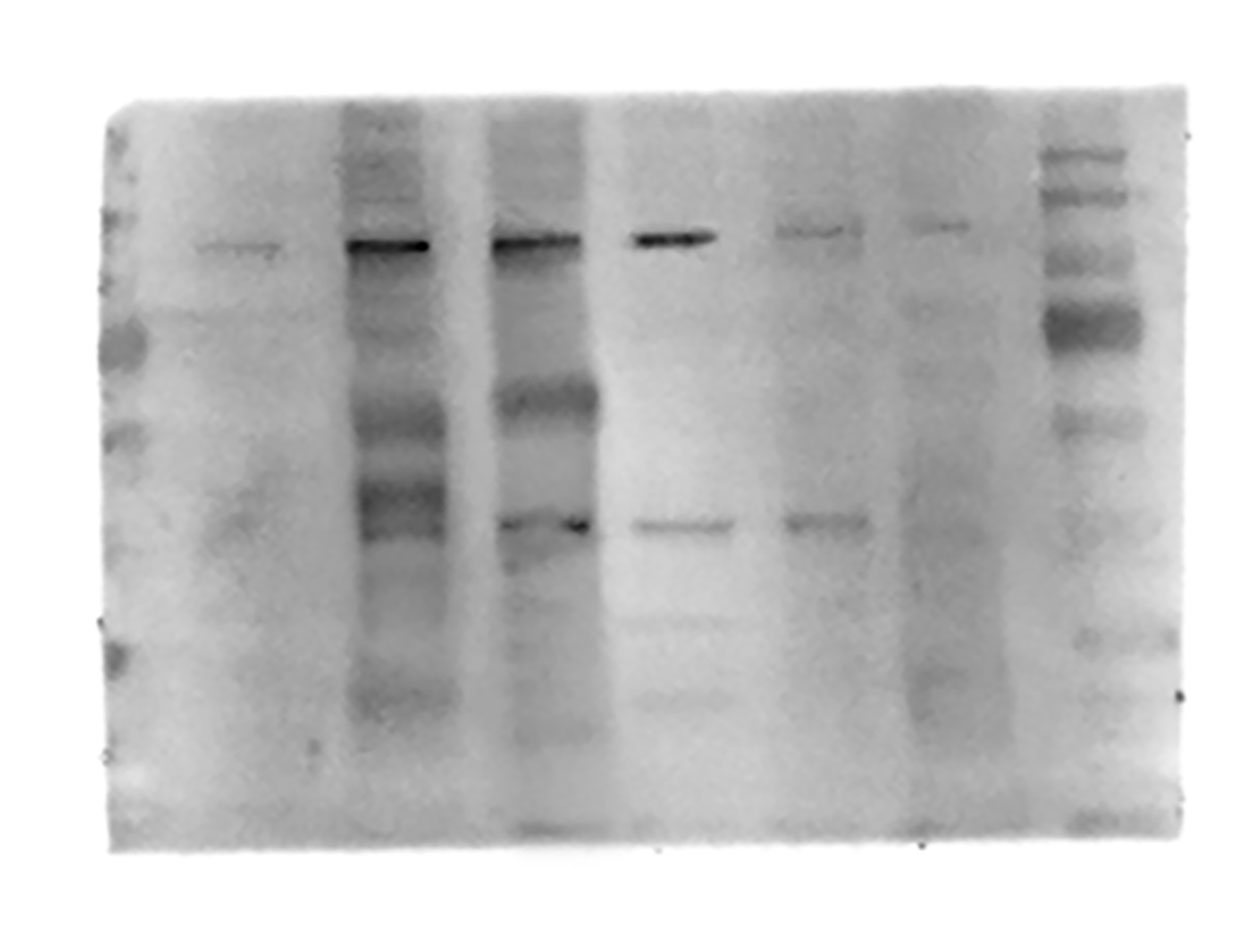

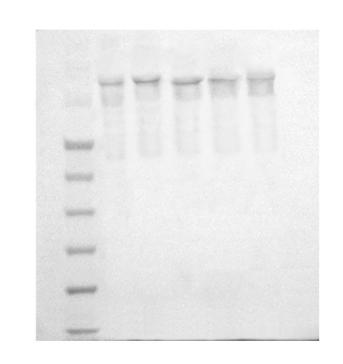

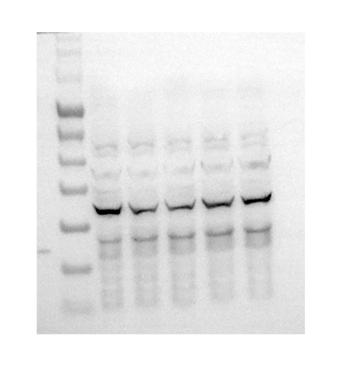

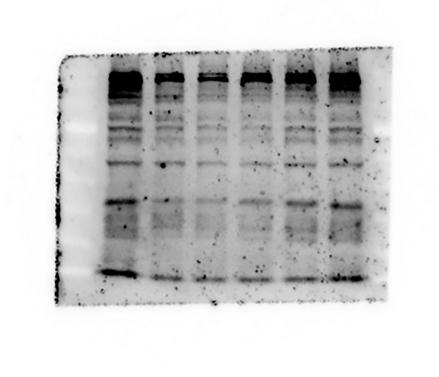

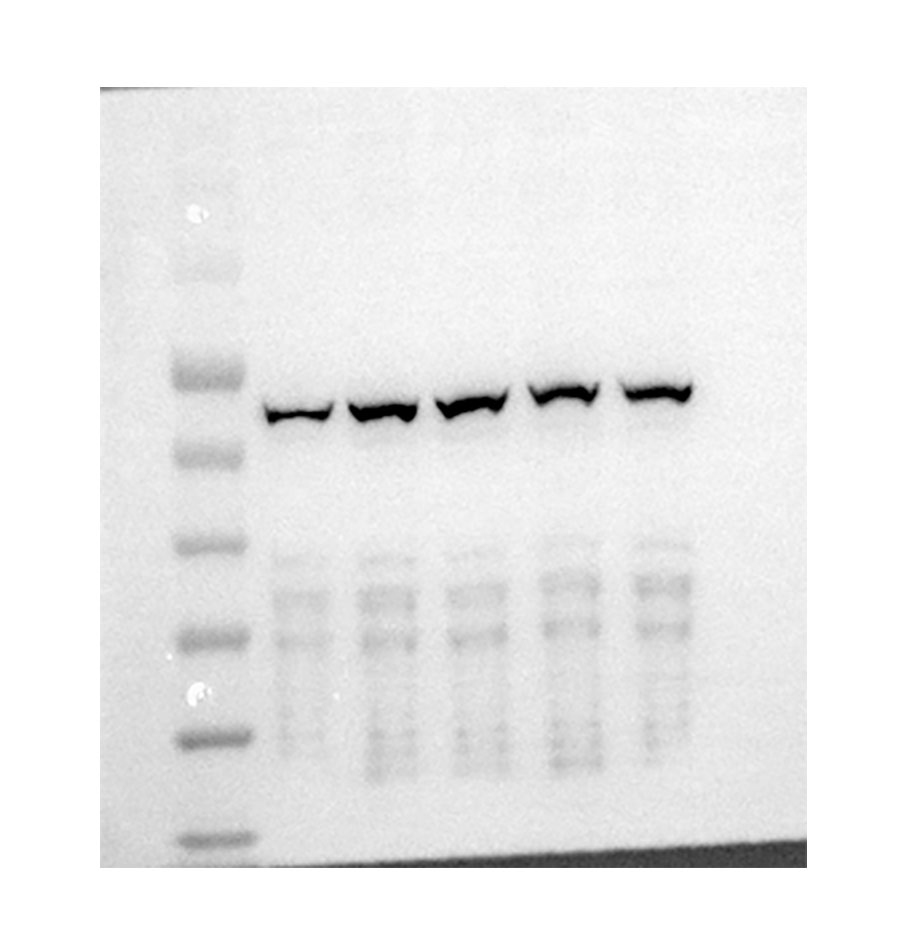

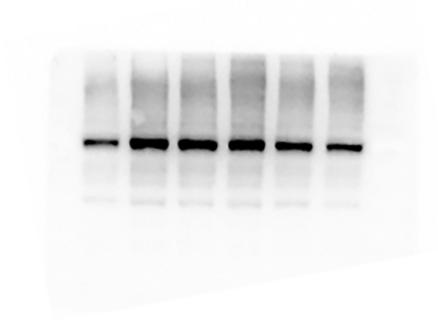

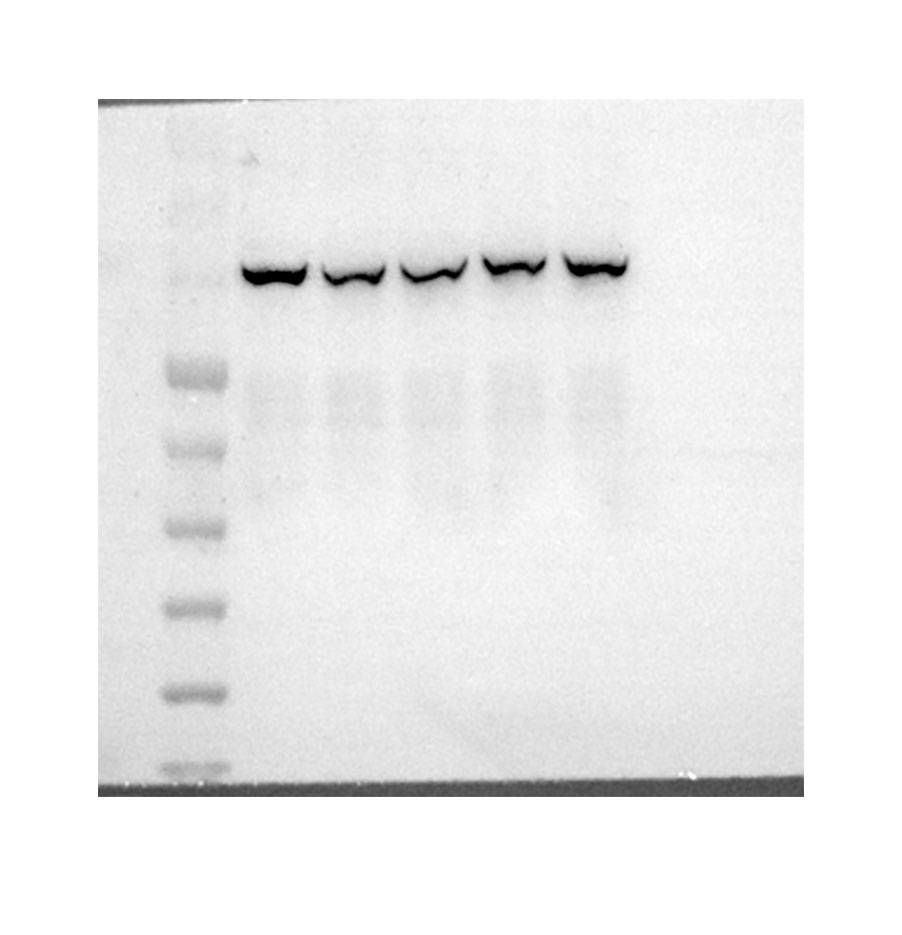

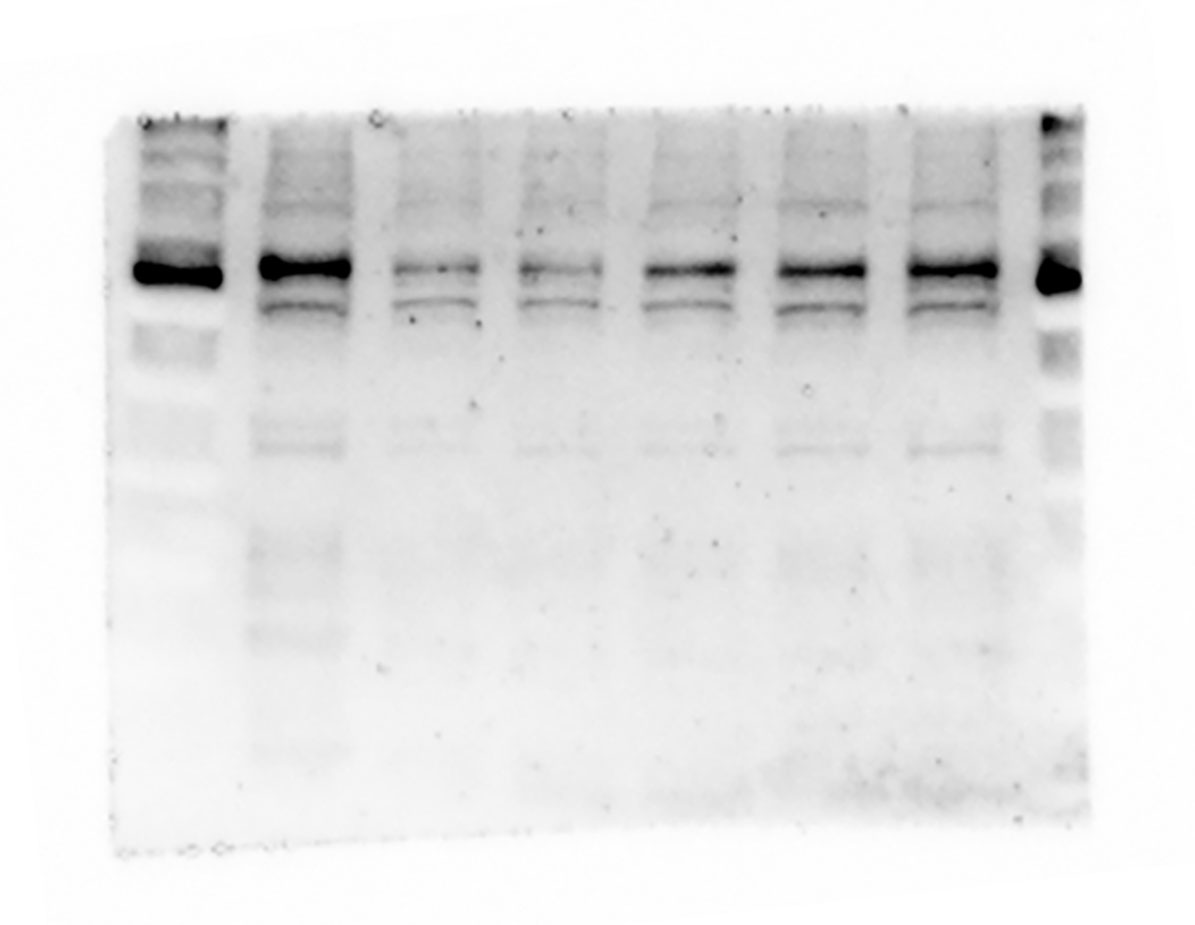

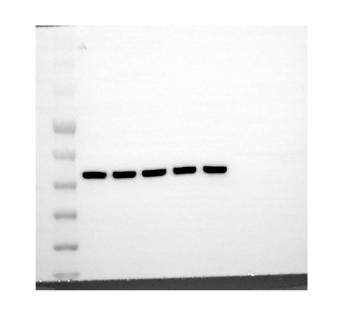

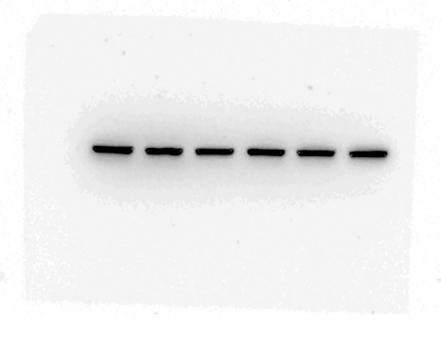


Nrf2

HO-1

BACH 1

HO-1(in vitro)

BACH1(in vitro)

β-actin

β-actin (in vitro)

Nrf2 (in vitro)

NF-κB

NF-κB (in vitro)
